# Primary Occipital Ewing's Sarcoma with Subsequent Spinal Seeding

**DOI:** 10.1155/2017/1521407

**Published:** 2017-06-13

**Authors:** Ali Alqahtani, Roaa Amer, Eman Bakhsh

**Affiliations:** ^1^College of Medicine, King Saud bin Abdulaziz University for Health Sciences, Riyadh, Saudi Arabia; ^2^Radiology Department, King Fahad Medical City, Riyadh, Saudi Arabia

## Abstract

Ewing's sarcoma is a primary bone cancer that mainly affects the long bones. This malignancy is particularly common in pediatric patients. Primary cranial involvement accounts for 1% of cases, with occipital involvement considered extremely rare. In this case study, primary occipital Ewing's sarcoma with a posterior fossa mass and subsequent relapse resulting in spinal seeding is reported. A 3-year-old patient presented with a 1-year history of left-sided headaches, localized over the occipital bone with progressive torticollis. Computed tomography (CT) imaging showed a mass in the left posterior fossa compressing the brainstem. The patient then underwent surgical excision followed by adjuvant chemoradiation therapy. Two years later, the patient presented with severe lower back pain and urinary incontinence. Whole-spine magnetic resonance imaging (MRI) showed cerebrospinal fluid (CSF) seeding from the L5 to the S4 vertebrae. Primary cranial Ewing's sarcoma is considered in the differential diagnosis of children with extra-axial posterior fossa mass associated with destructive permeative bone lesions. Although primary cranial Ewing's sarcoma typically has good prognosis, our patient developed metastasis in the lower spine. Therefore, with CNS Ewing's sarcoma, screening of the entire neural axis should be taken into consideration for early detection of CSF seeding metastasis in order to decrease the associated morbidity and mortality.

## 1. Introduction

Ewing's sarcoma is considered the second most common pediatric primary bone tumor, affecting mainly the long bones and pelvis. Primary cranial involvement accounts for approximately 1% of Ewing's sarcoma cases, with a primary occipital bone tumor being considered very rare [1–3]. Metastatic Ewing's sarcoma accounts for about 25% of cases [4]. In this report, a rare case of primary occipital Ewing's sarcoma with posterior fossa mass relapse as spinal seeding is presented.

## 2. Case Report

**Figure 1:**
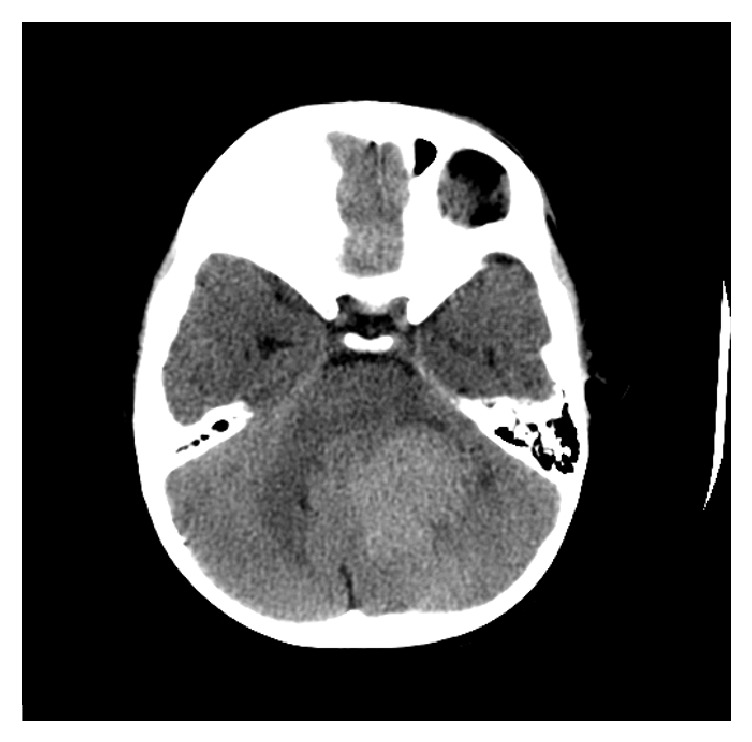
Axial CT of the brain revealed left-sided posterior fossa hyperdense mass compressing the fourth ventricle and brainstem.

**Figure 2:**
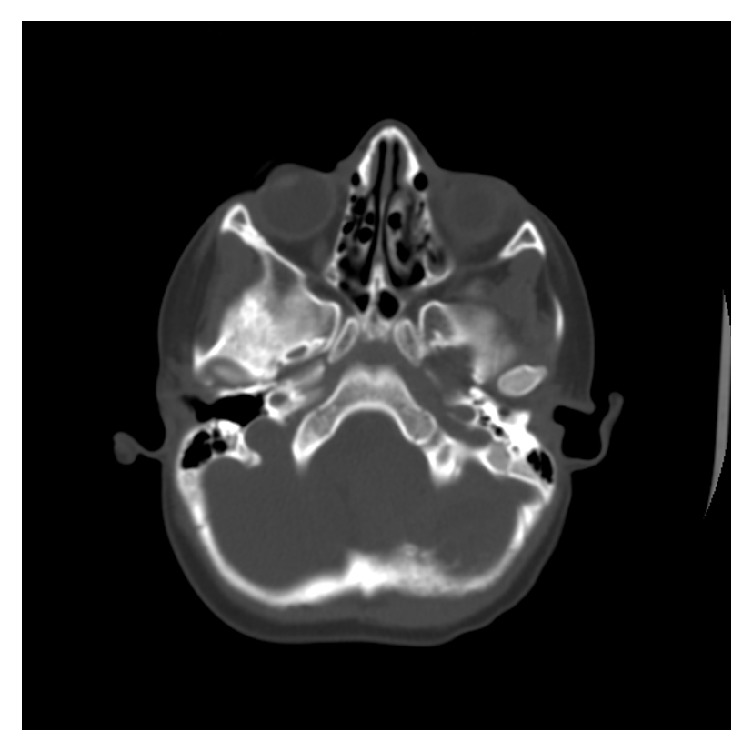
Axial CT scan bone window shows loss of the inner skull table and cortical outline with permeative bone reaction indicating bone destruction.

A 3-year-old male presented with a left-sided headache localized over the occipital bone, with progressive torticollis over the last year, as well as vomiting and ataxia over the last week. Examination of the patient was unremarkable with the exception of bilateral lower limb weakness. Computed tomography (CT) without contrast showed a large lobulated heterogeneous hyperdense lesion in the left posterior fossa. The lesion exerted a mass effect compressing the 4th ventricle and brainstem structures, with supratentorial triventricular hydrocephalus (Figure 1) accompanied by permeative bone reaction in the left occipital bone (Figure 2).

**Figure 3:**
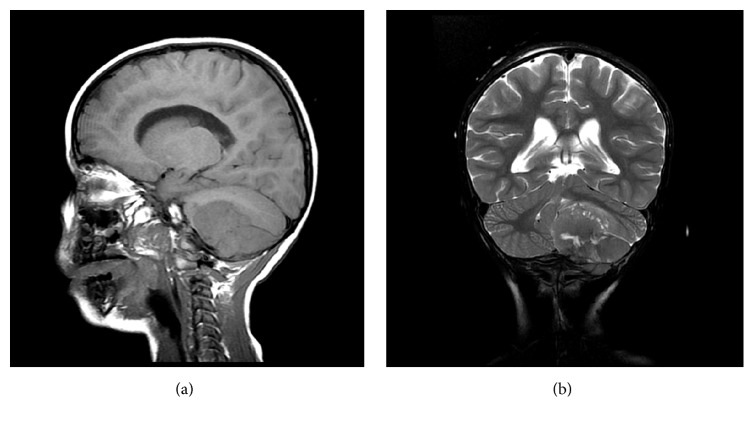
(a) Sagittal T1 weighted image of the brain showing extra-axial durally based solid mass compressing the cerebellum and showing loss of normal cortical bone intensity suggesting invasion of the marrow space. (b) Coronal T2 weighted images showing central necrosis of the mass and compression effect over the fourth ventricle.

**Figure 4:**
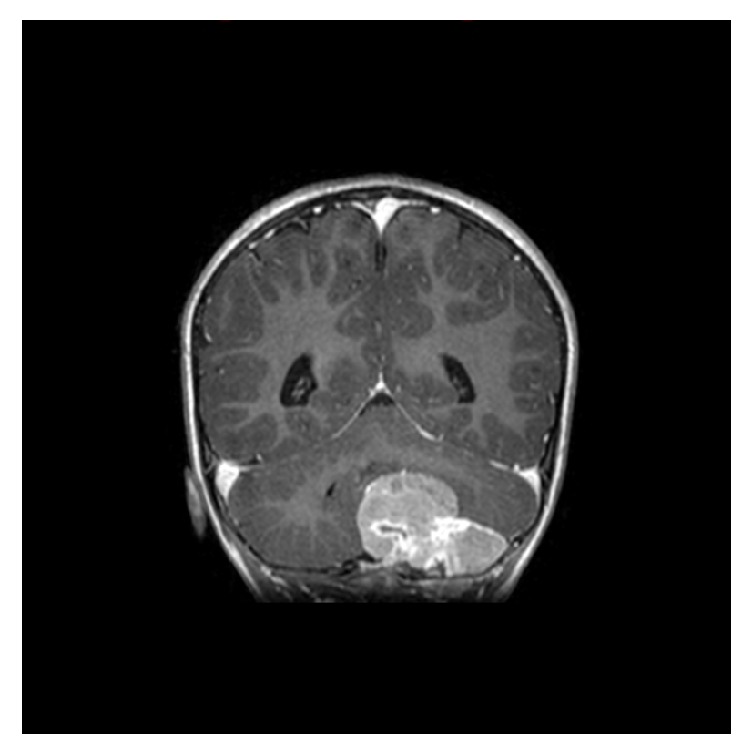
Coronal enhanced T1 weighted image showing intensely enhancing mass invading the skull table.

Magnetic resonance imaging (MRI) of the brain showed evidence of an infratentorial posterior fossa extra-axial mass that appeared to be dural. There was invasion of the marrow space of the left occipital bone (Figure 3(a)). In addition, heterogeneous signal intensity in T2-weighted images with multiple areas of central higher signal intensities representing necrosis was observed (Figure 3(b)). MRI with contrast enhancement showed the mass invading the inner skull table and intradiploic space (Figure 4).

Due to the primary malignancy, there was a significant mass effect over the left cerebellar hemisphere. The plane of cleavage between the tumor and the cerebellum was lost, indicating invasion. Furthermore, the tumor was noted to protrude through the foramen magnum, causing compression and displacement over the brainstem, especially over the upper medulla. Additional diagnostic testing with blood and imaging were found to be normal. The patient's tumor had destroyed the involved occipital bone, with minimal invasion of the dura and obvious calcification.

The patient underwent a left parietooccipital craniotomy for tumor resection, with samples collected for histopathological evaluation. The gross specimen had a size of 5 × 4 × 2 cm^3^, grey color, irregular, firm consistency, without obvious necrosis or hemorrhage, and confirmed cerebellar invasion. From histopathology, the tumor sheets showed a compactly arranged round cell tumor with cells having clear cytoplasm. Small spicules of bone were also seen in the tumor tissue, suggestive of occipital bone destruction. It was noted that the cells were large with irregular nuclear membranes and prominent nucleoli. The pleomorphic cells with few pseudorosettes were positive for EWS-FLI1 translocation in 98% of the cells. On immunohistochemistry, cells were immunoreactive for surface antigen CD99/MIC2 and positive for neuron-specific enolase and Leu 7.

**Figure 5:**
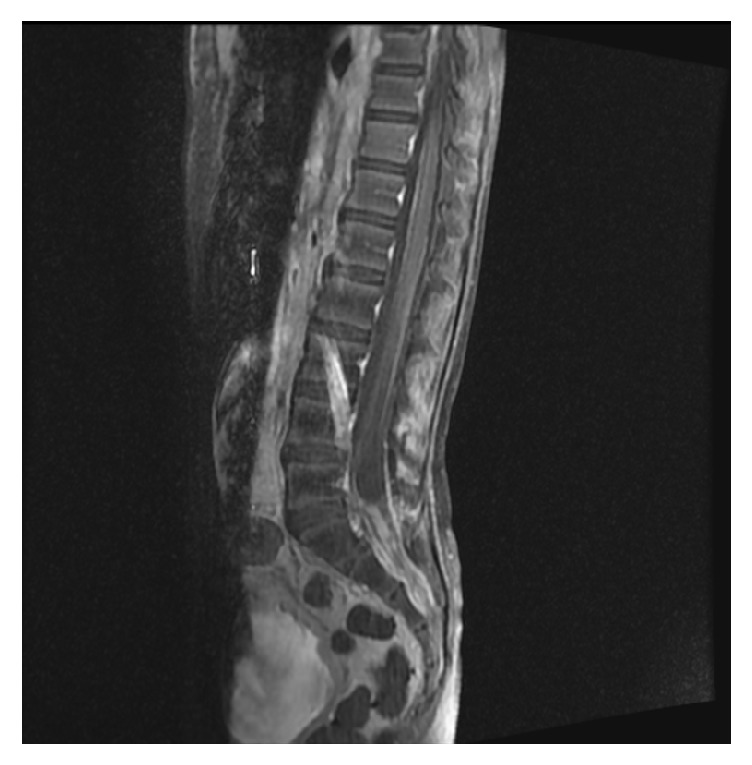
Contrast enhanced sagittal T1 weighted image of the lumbosacral spine showing an enhancing intradural mass extending from L5 to S1 eroding the posterior aspect of S2 denoting intraspinal CSF seeding.

Postoperative CT confirmed total tumor resection. The patient then received adjuvant chemotherapy with focal radiation. The patient was given vincristine, doxorubicin, and cyclophosphamide followed by ifosfamide and etoposide plus radiation to the left cerebellopontine angle. After three cycles of chemotherapy, a subsequent MRI showed marginal response. The patient was then switched to another chemotherapy regimen consisting of vincristine, temozolomide, and irinotecan. The patient was lost to follow-up examination for eight-month duration after confirmation of remission. The patient then presented with severe constant lower back pain and urinary incontinence during the day. A whole-spine MRI showed intraspinal enhancing lesions representing cerebrospinal fluid (CSF) seeding, extending from the level L5-S4 (Figure 5). Palliative treatment including spinal radiation and ICE (ifosfamide, carboplatin, and etoposide) chemotherapy protocol was administered. The patient has been followed for six months to date, with disease stability from the palliative treatment.

## 3. Discussion

Primary Ewing's sarcoma affecting the cranial bones accounts for about 1–9% of cases [1, 5, 6]. Most patients are affected at ages 5–30 years, with the highest peak occurring in the second decade of life, without predisposition for either sex. The most frequent sites in primary cranial Ewing's sarcoma are temporal followed by frontal. The most common presenting symptom is intermittent local pain. In addition, headache, vomiting, and papilledema are common symptoms. However, our patient presented with a unique finding of hydrocephalus manifesting as ataxia. Torticollis also presented in our patient, with this condition having been previously reported to be the first sign of a posterior fossa tumor [7]. From the previous study, the average duration from the onset of torticollis to diagnosis of Ewing's sarcoma was 9.6 weeks [7]. As our patient had a 1-year history of progressive torticollis prior to diagnosis, a delayed diagnosis of the primary tumor is reasonable.

Radiologically, Ewing's sarcoma shows bone destruction in a permeative pattern that may be accompanied with large soft tissue involvement, indicative of a highly aggressive tumor. Tumors involving the skull with adjacent soft tissue have a comprehensive differential diagnoses, which includes metastatic tumors such as neuroblastoma, lymphoma, and rhabdomyosarcoma and primary tumors such as meningiosarcoma and sarcomatous malignant lesions. Radiologically, neuroblastomas usually have hair-on-end periosteal appearance, osteolytic lesions, and separation of sutures [8]. Secondary skull lymphomas present as lesions extending to both the subcutaneous and epidural spaces, with meningeal penetration [9]. Rhabdomyosarcoma tumors tend to be isointense to muscle on T1-weighted images and high in intensity on T2-weighted images, with the orbit being the primary site in 90% of cases [10]. However, a case was reported with rhabdomyosarcoma in the occiput with grey color, compressing the brain tissue and thereby mimicking Ewing's sarcoma with no signs of calcification on imaging [11]. Our patient's CT and MRI showed intralesional-dense calcifications. Moreover, primary meningeal sarcoma tumors show cystic structures within the masses, heterogeneity on contrast enhancement, and connections to the meninges [12].

Our patient's imaging studies showed an extra-axial durally based left posterior fossa mass with CSF cleft eroding the inner skull table and involved intradiploic space with intralesional-dense calcifications and vivid intensity enhancement. The absent plane of cleavage between the medial border of the mass and the adjacent cerebellar tissue was highly indicative of invasion, which was eventually confirmed surgically. These findings explain the patient's clinical presentation.

The histopathology report revealed that the patient had a round cell tumor, which was positive for Ewing's sarcoma, EWS-FLI1 fusion gene, and membranous expression of CD99 and S-100 proteins. Round cell tumors can involve a variety of differentials including primitive neuroectodermal tumors such as neuroblastoma and Ewing's sarcoma, as well as other tumors such as lymphoma and rhabdomyosarcoma [13]. The EWS-FLI1 fusion gene can also be seen in tumors other than Ewing's sarcoma, such as neuroblastoma and rhabdomyosarcoma, giving the appearance of Ewing's sarcoma. Although unusual for Ewing's sarcoma, our patient's histopathology report showed large cells with irregular nuclear membranes and prominent nucleoli [13].

Ewing's sarcoma is treated with a multidisciplinary approach that includes surgery, chemotherapy, and radiotherapy. The patient underwent a gross total surgical resection of the tumor. Three cycles of adjuvant chemotherapy consisting of vincristine, doxorubicin, and cyclophosphamide, followed by ifosfamide and etoposide, were administered along with local radiotherapy. Owing to marginal tumor control, the regimen was switched to vincristine, temozolomide, and irinotecan. Postoperative imaging indicated remission. There was an eight-month period of loss of follow-up where the patient presented with severe constant lower back pain and urinary incontinence. Spinal MRI revealed intraspinal enhancing lesions extending from the L5-S4 vertebrae, and the patient was managed with palliative chemoradiation. The patient remained stable with Eastern Cooperative Oncology Group (ECOG) performance status of 3.

Female gender, the absence of systemic symptoms and metastasis at diagnosis, long duration of symptoms (more than six months), and peripheral tumor location are indicators of favorable prognosis [14]. Despite our patient having a 1-year duration of symptoms and absence of metastasis at diagnosis, this was not the patient's reported experience. Appreciating symptoms such as headaches is difficult in a 3-year-old patient, hence the delayed diagnosis. Moreover, there was a period of loss of follow-up after remission, which could be a contributing factor to the recurrence and metastasis.

## 4. Conclusion

Primary cranial Ewing's sarcoma should be considered in the differential diagnosis of pediatric extra-axial durally based posterior fossa masses, particularly when associated with involvement of the adjacent bone. To improve prognosis, Ewing's sarcoma of the CNS should have screening of the entire neural axis for early detection of CSF seeding metastasis.
